# Auricular Vagus Nerve Stimulation for Gastrointestinal Disorders: Hope or Hype?

**DOI:** 10.1002/ueg2.12751

**Published:** 2025-01-02

**Authors:** Daniel Keszthelyi

**Affiliations:** ^1^ Department of Gastroenterology‐Hepatology Maastricht University Medical Center+ Maastricht the Netherlands

The outer ear has been a site of therapeutic interest since early human civilization: women in ancient Egypt who did not want any more children, had their external ear pricked with a needle or cauterized with heat [1]. It was not until 1831 though, when the German anatomist Friedrich Arnold (1803–1890) provided a systematic theoretical explanation for bodily reactions to ear stimulation by describing that irritation of the posterior wall of the external acoustic meatus elicited coughing (the so‐called Arnold's reflex). He attributed this reflex to the auricular branch of the vagus nerve, which has since been referred to as Arnold's nerve [2].

The potential relevance of the auricular branch of the vagus nerve in gastrointestinal function is suggested by its alternative eponym: Alderman's nerve. This is a centuries‐old reference to the Aldermen of the City of London and their practice of using rosewater bowls at ceremonial banquets generally associated with heavy eating. The banquet attendees were encouraged to place a cold table napkin moistened with rosewater behind their ears with the belief that this promoted gastric emptying and aided digestion [3].

The modern concept of transcutaneous auricular vagus nerve stimulation (taVNS) was introduced more than 20 years ago as a non‐invasive alternative to surgical vagus nerve stimulation [4], which utilizes electrode cuffs wrapped directly around the left cervical bundle of the vagus, connected to an implanted pulse generator in the chest wall. This form of invasive vagus nerve stimulation was developed in the 1980s in patients with intractable epilepsy, and is approved for the treatment of refractory epilepsy and depression.

The non‐invasive nature of taVNS makes it attractive for the treatment of gastrointestinal disorders, in particular within the realm of neurogastroenterology. TaVNS is assumed to exert neuromodulatory effects by recruiting sensory afferent fibers and thus mimicing sensory input to the brainstem, more specifically the nucleus of the solitary tract [5], where other sensory afferents from the gut also terminate, which highlights the relevance of taVNS for disorders of gut‐brain interaction. The interest for auricular vagus nerve stimulation within neurogastroenterology has taken off sinds the landmark study in 2017 by Kovacic et al. in adolescents with chronic abdominal pain, mostly irritable bowel syndrome and functional abdominal pain syndrome [6].

In this issue of the United European Gastroenterology Journal, Liu et al. [7] describe a randomized controlled trial of 106 patients with chronic constipation (Rome IV) recruited from 5 tertially referral centers in China. Patients were treated with taVNS (stimulation to the left tragus) or sham (stimulation of the left earlobe, not innervated by the vagus) for 30 min, twice a day for a period of 4 weeks. The study was terminated prematurely due to lack of efficacy: response rate (defined as the proportion of patients with a weekly complete spontaneous bowel movement of 3 or more) was 17% for tVNS versus 19% in the sham group.

While this trial is indeed negative, a number of methodological aspects need mentioning. First, since the vagus nerve is believed to provide innervation up to the splenic flexure of the colon [8], the question arises whether the apparent lack of efficacy of taVNS in chronic constipation is related to the absence of vagal innervation in the very distal parts of the colon. In addition, the celiac vagal branches innervating the small and large intestine are believed to largely be derived from the right (and not the left) vagus [8]. Second, the use of a stimulation frequency of 25 Hz and the length of the treatment period of 4 weeks might not have been sufficient to reach a therapeutic effect. Third, sham stimulation at a different anatomical location without vagal innervation (such as the earlobe) can be considered a risk for deblinding in the setting of taVNS given the broad availability of information on this treatment entity in lay media.

Surely this is not the last we will hear of taVNS. In fact, vagus nerve stimulation has been described as the number 1 wellness trend in 2024 [9]. But before we can move beyond a mere health trend, we will first need to enhance our understanding of taVNS, define the most‐optimal treatment setting, and confirm therapeutic efficacy using high‐quality clinical trials.

## Conflicts of Interest

D.K. has received research funding from Rome Foundation, ZonMw, Horizon 2020, Horizon Europe, UEG, Dutch Foundation for Gastroenterology.

## Data Availability

The author has nothing to report.
